# The ND10 Complex Represses Lytic Human Herpesvirus 6A Replication and Promotes Silencing of the Viral Genome

**DOI:** 10.3390/v10080401

**Published:** 2018-07-29

**Authors:** Anirban Sanyal, Nina Wallaschek, Mandy Glass, Louis Flamand, Darren J. Wight, Benedikt B. Kaufer

**Affiliations:** 1 Institut für Virologie, Freie Universität Berlin, Robert von Ostertag-Straße 7-13, 14163 Berlin, Germany; a.sanyal@fu-berlin.de; 2 Institute for Molecular Infection Biology, Julius-Maximilians-Universität Wϋrzburg, 97080 Wϋrzburg, Germany; nina.wallaschek@uni-wuerzburg.de; 3 School of Science and Sport, University of the West of Scotland, ML3 0JB Glasgow, UK; Mandy.Glass@uws.ac.uk; 4 MRC University of Glasgow Centre for Virus Research, G61 1QH Glasgow, UK; 5 Division of Infectious Disease and Immunity, CHU de Québec Research Center, Quebec City, QC G1V 4G2, Canada; louis.flamand@crchudequebec.ulaval.ca; 6 Department of Microbiology, Infectious Disease and Immunology, Faculty of Medicine, Université Laval, Quebec City, Québec G1V 0A6, Canada

**Keywords:** human herpesvirus 6, ND10 complex, PML, lytic replication, latency

## Abstract

Human herpesvirus 6A (HHV-6A) replicates in peripheral blood mononuclear cells (PBMCs) and various T-cell lines in vitro. Intriguingly, the virus can also establish latency in these cells, but it remains unknown what influences the decision between lytic replication and the latency of the virus. Incoming virus genomes are confronted with the nuclear domain 10 (ND10) complex as part of an intrinsic antiviral response. Most herpesviruses can efficiently subvert ND10, but its role in HHV-6A infection remains poorly understood. In this study, we investigated if the ND10 complex affects HHV-6A replication and contributes to the silencing of the virus genome during latency. We could demonstrate that ND10 complex was not dissociated upon infection, while the number of ND10 bodies was reduced in lytically infected cells. Virus replication was significantly enhanced upon knock down of the ND10 complex using shRNAs against its major constituents promyelocytic leukemia protein (PML), hDaxx, and Sp100. In addition, we could demonstrate that viral genes are more efficiently silenced in the presence of a functional ND10 complex. Our data thereby provides the first evidence that the cellular ND10 complex plays an important role in suppressing HHV-6A lytic replication and the silencing of the virus genome in latently infected cells.

## 1. Introduction

Human herpesvirus-6 (HHV-6) is a betaherpesvirus that has been classified as two distinct virus species, HHV-6A and HHV-6B, due to differences in their biological and genetic characteristics [1,2,3]. Primary infection with HHV-6B causes roseola infantum (sixth disease), a febrile illness in children that is occasionally accompanied by neurological problems like seizures and encephalitis [4,5,6]. The clinical manifestations and epidemiology associated with HHV-6A infections are currently less well understood. Primary infection is followed by a lifelong persistence in infected individuals, termed latency [7]. Both viruses have been shown to integrate their genome into host telomeres of latently infected cells [8,9]. Integration also occurs in germ cells, resulting in individuals that harbor the integrated virus in every single cell in their body [10,11]. This condition is termed inherited chromosomally integrated HHV-6 (iciHHV-6) and is present in about 1% of the human population [12]. HHV-6A/B can reactivate from latently infected cells, as well as in iciHHV-6 patients, which is associated with several diseases including encephalitis and graft rejection following transplantation [13,14].

HHV-6A/B replicate efficiently in human cord blood mononuclear cells (CBMC) and peripheral blood mononuclear cells (PBMC) as well as in various T-cell lines, including JJHan and SupT1 cells [14]. Intriguingly, the viruses can also establish latency in these cell lines, but it remains unknown how the decision between lytic replication and latency is made. In the target cells, the incoming virus genome is confronted with the nuclear domain 10 (ND10) complex that possesses antiviral activity against a plethora of viruses [15,16,17]. The ND10 complex has three key constituents, namely, promyelocytic leukemia antigen (PML), speckled protein of 100 kDa (Sp100), and human death domain-associated protein 6 (hDaxx). PML is crucial for the ND10 complex as it is required for the proper localization of other ND10-associated proteins [18]. Several herpesvirus proteins have been shown to manipulate these components and disrupt the ND10 complex during the establishment of infection [19,20]. For example, ICP0 of herpes simplex virus 1 (HSV-1) induces degradation of PML and Sp100 [21,22,23,24]. Similarly, the viral immediate early protein-1 (IE1) of human cytomegalovirus (HCMV) interacts with PML and induces dissociation of the ND10 complex [25,26,27]. In addition, HCMV pp71 induces degradation of hDaxx, a crucial step for productive HCMV gene expression [28,29,30]. Most known human herpesviruses have been found to efficiently subvert the ND10 complex to successfully establish lytic infection in the host, but the role of the ND10 complex in HHV-6 infection remains poorly understood.

In this study, we investigated the role of the ND10 protein complex during HHV-6A infection. Immunofluorescence studies revealed that ND10 bodies are not dissociated, but their number is reduced in lytically infected cells. To address the role of the ND10 complex, we knocked down the key constituents PML, Sp100, and hDaxx in HHV-6A permissive cells using shRNAs. Lytic replication of HHV-6A was significantly enhanced upon knockdown of the ND10 complex. In addition, viral gene expression was more efficient in cells upon knockdown of ND10 complex. Our data provides evidence that ND10 complex suppresses HHV-6A replication and plays an important role in silencing the viral genome.

## 2. Materials and Methods 

### 2.1. Cells and Viruses 

JJHan and 293T cells were cultured in RPMI and DMEM media, respectively. Both media were supplemented with 10% FBS and 1% penicillin/streptomycin. Cells were grown at 37 °C, under 5% CO_2_ atmosphere. Recombinant HHV-6A (U1102 strain), expressing GFP under the control of the HCMV major immediate early (IE) promoter, was propagated on the human T cell line JJHan (HHV-6A-GFP) as described previously [31]. We generated a late gene reporter virus. GFP was fused with a P2a ribosome skipping motif to the major capsid protein U57 in pHHV-6A (vU57-P2A-GFP), an infectious BAC clone of HHV-6A (strain U1102) using two-step Red-mediated mutagenesis as described previously [32]. Recombinant clones were confirmed by restriction fragment length polymorphism (RFLP) and Sanger sequencing. Primers used for the mutagenesis and DNA sequencing are listed in Table 1.

### 2.2. Immunofluorescence 

To assess the effect of HHV-6A infection on the ND10 complex, JJHan cells were infected with HHV-6A-GFP. Cells were fixed with 4% paraformaldehyde (PFA) at 24 h post infection (hpi), permeabilized with 0.1% Triton-X 100 and blocked with 10% BSA. Cells were then stained with rabbit anti-PML (Bethyl Laboratories, Montgomery, TX, USA) and mouse anti-gp82 antibodies (clone 2-D6; HHV-6 foundation repository) at 1:1000 dilutions and further stained using goat anti-rabbit Alexa 568 and goat anti-mouse Alexa 647 antibodies, respectively. Images were acquired on an Andor iXon888 EMCCD using a Nikon-based spinning-disk confocal microscope at 100× magnification (Visitron Systems GmbH, Puchheim, Germany). Images were processed by ImageJ, Adobe Photoshop and Illustrator software (San Jose, CA, USA). One hundred cells were imaged and the number of PML foci per nucleus counted in a blinded manner. The HHV-6A infected cells were further grouped with respect to the stage of infection: (i) immediate early infected cells that only express GFP and (ii) GFP/gp82 double positive indicative of late replication. In addition, immunofluorescence was also used to confirm the knockdown of PML in JJHan cells after lentiviral transduction as described above. 

### 2.3. Lentivirus Production and Transduction 

To knockdown PML, hDaxx, and Sp100, lentiviruses were prepared by co-transfection of 293T cells with a pLKO-shDPS vector, containing shRNAs against PML, hDaxx, and Sp100 and the packaging plasmids pCMV-VSV-G and pCMV-dR8.91, as described previously [33]. JJHan (after called JJHan-KD) and 293T cells (after called 293T-KD) were transduced with these lentiviruses, selected using puromycin and single cell clones generated.

### 2.4. Western Blotting 

2 × 10^5^ JJHan-KD, 293T-KD, and corresponding control cells were harvested and lysed in a radioimmunoprecipitation assay (RIPA) buffer as described previously [34]. Proteins were separated on 12% SDS polyacrylamide gels, blotted onto nitrocellulose membranes, blocked and stained with the rabbit anti-PML (Bethyl Laboratories) followed by a HRP-conjugated goat anti-rabbit antibody and the Amersham™ ECL™ Prime western blotting detection kit (GE Healthcare, Chicago, IL, USA). Subsequent experiments were conducted on JJHan clone 6 or 293T-KD clone 2 cell lines.

### 2.5. CellVue Infection Assay 

To determine if the replication properties of HHV-6A are altered upon knockdown of the ND10 complex, we established a simple infection assay using the CellVue Claret far-red fluorescent membrane dye (Sigma-Aldrich, St. Louis, MO, USA). 2.5 × 10^5^ HHV-6A-GFP infected JJHan cells were stained with the CellVue dye and co-cultured with 1 × 10^6^ unstained JJHan or JJHan-KD target cells. Cells were then analyzed by FACS to assess the spread of HHV-6A to the uninfected (CellVue negative cells) 5 days post infection. In addition, CellVue negative cells were sorted and HHV-6A genome copies quantified by qPCR as described below. For FISH analyses, GFP positive CellVue negative cells (GFP+ CellVue/APC−) were isolated by FACS to obtain a pure infected target population. 

### 2.6. Quantification of HHV-6A Genome Copies 

DNA was isolated from sorted CellVue negative cells and mock infected cells using the RTP DNA/RNA Virus Mini Kit (Stratec, Birkenfeld, Germany) according to manufacturer’s instructions. To determine HHV-6A genome copies, qPCR was performed using specific primers and probes for the HHV-6A U86 gene as described previously [31]. U86 genome copies were normalized against cellular beta-2 microglobulin (B2M). Primers and probe sequences are listed in Table 1.

### 2.7. Fluorescence In Situ Hybridization (FISH) 

To determine the number of lytically infected cells, a pure target population was obtained as described above and analyzed by FISH to visualize lytic replication foci. Interphase nuclei were analyzed by FISH as described previously using an Axio Imager M1 fluorescence microscope (Zeiss, Oberkochen, Germany) [31,35,36,37]. Images of one hundred cells for each group were taken in a blinded manner, the number of cells containing lytic HHV-6A replication centers was quantified and the percentage of lytically infected cells calculated.

### 2.8. Late Gene Reporter Assay

To confirm that the ND10 complex suppresses late gene expression, the late gene reporter virus BAC clone was transfected into 293T or 293T-KD cells by PEI transfection. U57-GFP expression was analyzed by FACS 5 days post transfection.

### 2.9. Statistical Analyses

Statistical analyses were performed using GraphPad Prism5. Flow cytometry, qPCR, and FISH data were analyzed using a Mann–Whitney U test.

## 3. Results

### 3.1. Analysis of ND10 Complex in HHV-6A Infected Cells

**Figure 1:**
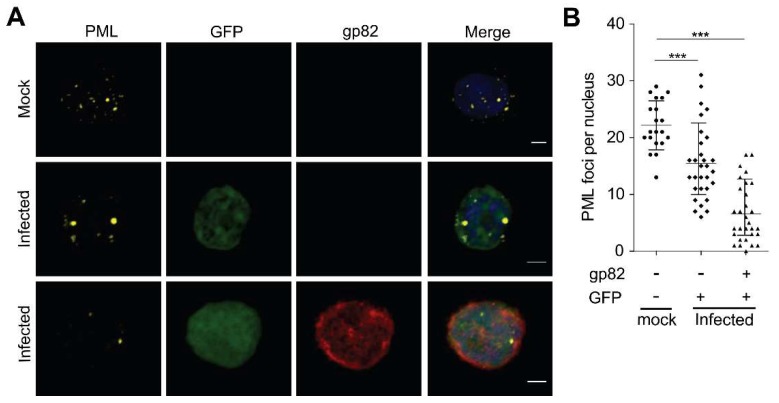
HHV-6A infection reduces the number of PML bodies. (**A**) HHV-6A-GFP and mock infected JJHan cells were immunostained for PML (yellow) and gp82 (red; late viral gene) and analyzed by confocal microscopy. Virus infected cells could be identified by GFP (green) and nuclei were stained with DAPI (blue). Representative images are shown for HHV-6A-GFP and mock infected cells. The scale bars correspond to 3 μm. (**B**) Quantification of PML foci in the nucleus of HHV-6A-GFP and mock infected JJHan cells (*n* = 100). Infected cells were grouped by the stage of infection, where GFP is expressed during the early stage of infection, while both GFP and gp82 are expressed during late lytic replication. Results are shown as the mean of three independent experiments with standard errors (***, *p* < 0.001).

Many herpesviruses induce the degradation of PML, resulting in the dissociation of the ND10 complex, which allows them to overcome its antiviral activity. To determine if HHV-6A also disrupts the ND10 complex, we analyzed cells infected with HHV-6A-GFP, expressing GFP under the control of the HCMV major immediate early promoter. PML staining revealed that the ND10 complex was not dissociated during HHV-6A infection (Figure 1A, Figure S1). However, the number of PML foci in the nucleus of infected cells was significantly reduced compared to uninfected cells (Figure 1B). This reduction was more prominent in cells expressing the late gene product gp82, indicative of a late phase of lytic replication, compared to cells expressing only GFP, driven by the immediate early promoter. In addition, the PML foci appeared larger in infected cells than in uninfected control cells (Figure 1A). In contrast to other human herpesviruses, that disrupt ND10 to promote virus replication, our data shows that HHV-6A does not disrupt the anti-viral ND10 complex.

### 3.2. Generation and Analysis of ND10 Knockdown Cells

**Figure 2:**
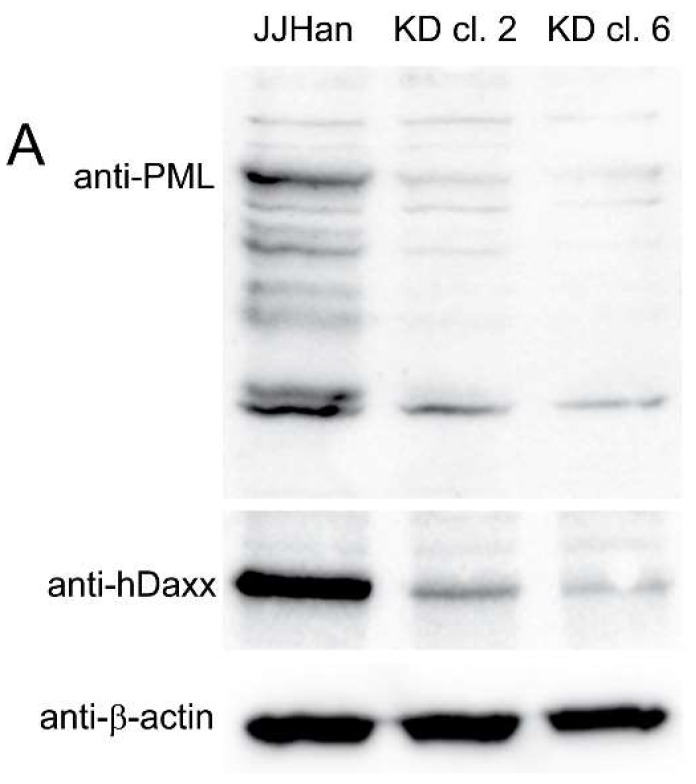

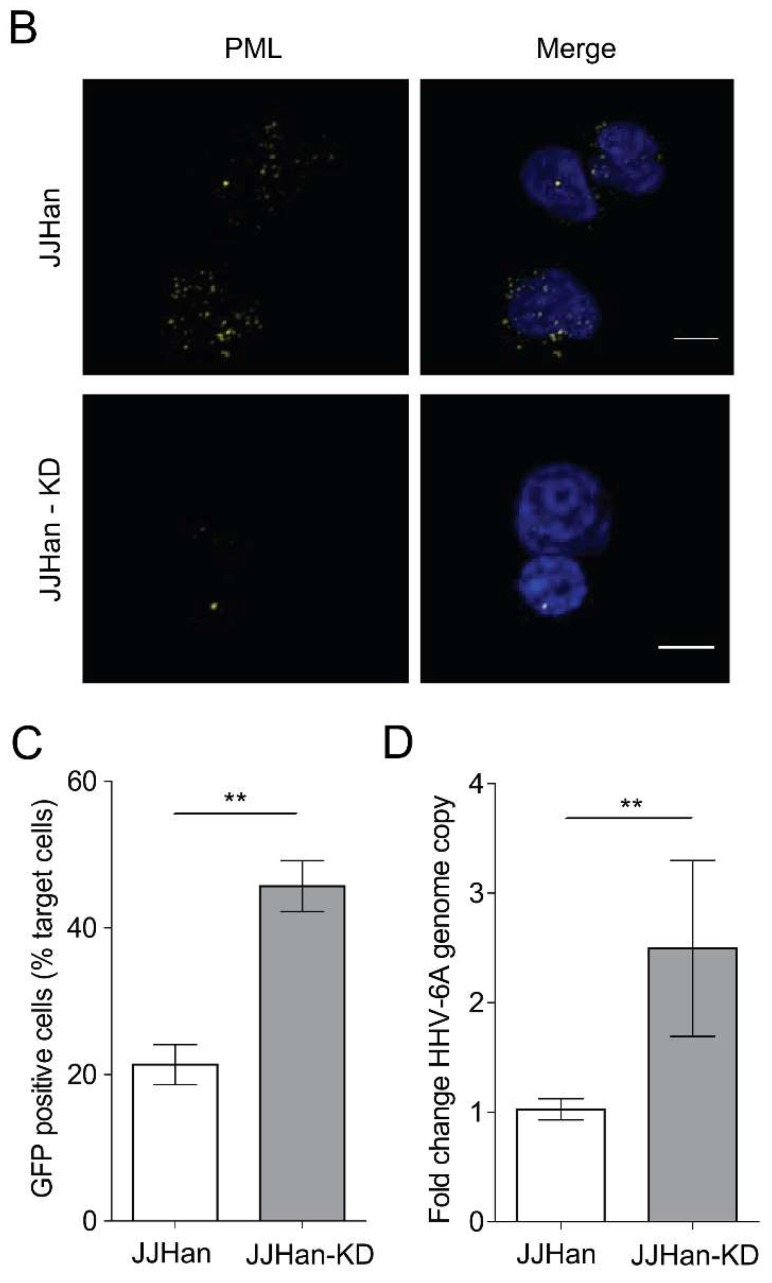
Effect of ND10 complex knockdown in JJHan cells on HHV-6A replication. (**A**) PML and hDaxx knockdown were assessed in two independent knockdown JJHan clones by western blotting. (**B**) PML knockdown was confirmed in JJHan clone 6 by indirect immunofluorescence against PML protein (yellow). Representative images are shown where the nuclei were stained with DAPI (blue) (scale bars correspond to 3 μm). (**C**) Flow cytometry analysis to quantify the number of GFP expressing cells upon infection of JJHan or JJHan-KD clone 6. Results are shown as the mean of three independent experiments with standard errors (**, *p* < 0.01). (**D**) qPCR analysis to determine the HHV-6A genome copies in infected JJHan and JJHan-KD clone 6 cells. Results are shown as the mean of five independent experiments with standard errors (**, *p* < 0.01).

To determine if the ND10 complex inhibits HHV-6A replication, we knocked down its major components PML, hDaxx, and Sp100 in JJHan cells (JJHan-KD) using a previously published single shRNA lentivirus vector [33]. Transduction of cells with this lentivirus has previously been shown to produce a stable knockdown of PML, hDaxx, and Sp100 [33]. We confirmed the knockdown of PML by western blotting and immunofluorescence (Figure 2A,B), indicative of the loss of all three ND10 proteins [33]. To determine the effect of the ND10 knockdown on HHV-6A replication, we infected JJHan-KD clone 6 and control cells with HHV-6-GFP. FACS analysis revealed that the percentage of infected cells was significantly increased in the absence of the ND10 complex (Figure 2C). To quantify virus replication, we performed qPCR and found that HHV-6A genome copies were also significantly increased upon ND10 knockdown (Figure 2D). Therefore, removal of the ND10 complex led to more efficient HHV-6A lytic replication.

### 3.3. Effect of the ND10 Complex on HHV-6A Replication

**Figure 3:**
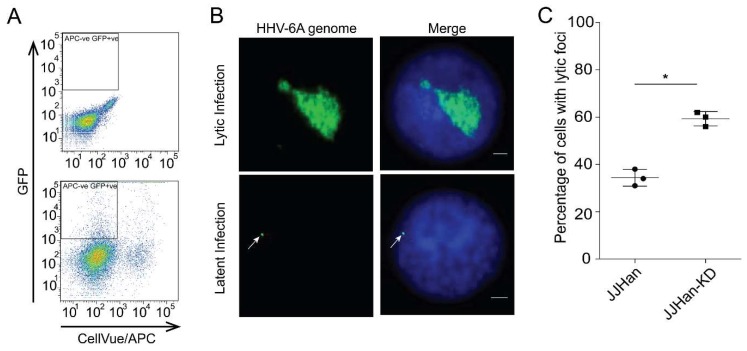
Quantification of lytic replication in JJHan-KD cells. (**A**) Gating strategy to isolate pure HHV-6A infected cell population. Infected GFP positive target cells were sorted and subsequently analyzed by FISH. (**B**) Representative FISH images showing the HHV-6A genome (green) in interphase nuclei (DAPI, blue) in lytically and latently infected cells. In latently infected cells, the viral genome is indicated with an arrow (scale bars correspond to 3 μm). (**C**) The percentage of lytically infected cells was quantified in JJHan and JJHan-KD cells (*n* = 100) in a blinded manner. Results are shown as the mean of three independent experiments with standard errors (*, *p* < 0.05).

HHV-6A can efficiently replicate in JJHan cells, but also establishes latency in these cells [31]. To determine if the ND10 complex influences this decision, we stained HHV-6A-GFP infected JJHans with a membrane dye (CellVue) and co-cultured them with uninfected and unstained JJHans. We then sorted a pure population of newly infected target cells (GFP+ Cellvue-; Figure 3A) and determined the percentage of lytically infected cells by fluorescence in situ hybridization (FISH) (Figure 3B). Notably, the percentage of lytically infected cells was significantly increased in the absence of the ND10 complex (Figure 3C).

### 3.4. ND10-Mediated Suppression of Viral Protein Expression in Latently Infected Cells 

**Figure 4:**
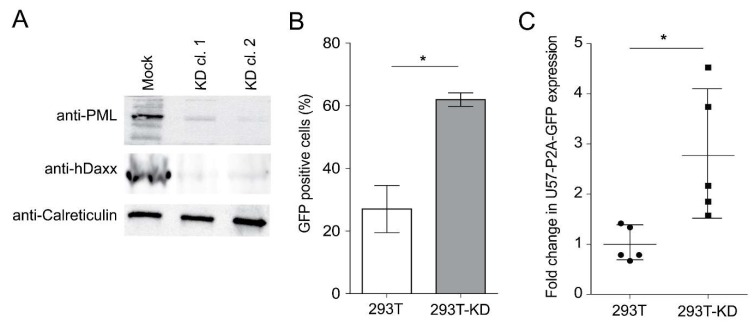
Depletion of ND10 components in 293T cells and its effects on HHV-6A gene expression. (**A**) PML and hDaxx knockdown was assessed in two independent 293T knockdown cell clones by western blotting. (**B**) Quantification of the GFP expression in 293T and 293T-KD clone 2 cells at three days = post-transfection with the HHV-6A-GFP BAC by FACS. Results are shown as the mean of three independent experiments with standard errors (*, *p* < 0.05). (**C**) Quantification of the expression of the major capsid protein U57 (late gene) by FACS in 293T and 293T-KD clone 2 cells five days post HHV-6A-U57-p2a-GFP BAC transfection by FACS. Results are shown as the mean of three independent experiments with standard errors (*, *p* < 0.05).

To determine if the presence of the ND10 complex contributes to HHV-6A genome silencing, we assessed viral protein expression in 293T cells, which are used to assess HHV-6 integration and only allow limited virus replication. We knocked down the three ND10 components in 293T cells (293T-KD) and confirmed the knockdown of PML by western blotting and immunofluorescence (Figure 4A). 293T-KD clone 2 and control cells were transfected with the HHV-6A-GFP genome and expression of GFP was analyzed by FACS three days post transfection. The number of expressing GFP cells was significantly higher upon knockdown of the ND10 components (Figure 4B). To confirm that HHV-6A genes are also suppressed by the ND10 complex, we used a late gene reporter virus genome expressing GFP fused to the late major capsid protein U57 via a P2a ribosome skipping motif (HHV-6A-U57-p2a-GFP, Figure S2). The HHV-6A-U57-p2a-GFP genome was transfected into 293T-KD and control cells and the percentage of U57-GFP expressing cells was analyzed by FACS five days post transfection. The number of U57-GFP expressing cells was significantly increased upon knockdown of the ND10 components (Figure 4C). Taken together, our data show that the ND10 complex contributes to suppression of genes in the HHV-6A genome.

## 4. Discussion

Herpesviruses have evolved mechanisms to overcome the antiviral activities of the ND10 complex. We observed that HHV-6A does not dissociate the ND10 complex during lytic replication, unlike other herpesviruses [19,20]. The number of ND10 foci in the nucleus of infected cells was significantly reduced compared to uninfected cells and the foci appeared slightly larger. Similar observations have also been made for HHV-6B [38], suggesting that these closely related betaherpesviruses do not counteract ND10 in the same manner as HCMV and other herpesviruses. 

PML, hDaxx, and Sp100 have previously been shown to be involved in heterochromatinization and chromatin condensation [39,40], suggesting that they could potentially be involved in the direct silencing of the HHV-6A genome and suppression of lytic replication. In the case of HCMV, PML, and hDaxx have been shown to induce a transcriptional repression of HCMV immediate early genes in non-permissive cell lines [41]. Sp100 interacts with the heterochromatin protein 1 (HP1) [15,39] and associates with unmethylated CpG DNA [15,42]. In addition, the Sp100B isoform can act as a transcriptional repressor for both cellular and viral promoters [43]. Therefore, PML, hDaxx, and Sp100 could indeed induce silencing of the HHV-6A genome, resulting in a quiescent infection rather than lytic replication. 

To determine if the ND10 complex suppresses HHV-6A replication, we knocked down the expression of PML, hDaxx, and Sp100 using an shRNA vector developed by Glass and colleagues [33]. Using this system, we were able to successfully knockdown the expression of ND10 complex in JJHan (JJHan-KD) and 293T cells (293T-KD). Infection of JJHan-KD cells revealed that HHV-6A replicated more efficiently compared to the parental cell line by FACS and qPCR. In addition, we isolated infected cells by FACS and could demonstrate that the number of lytically infected cells increased upon knockdown of the ND10 components, suggesting that less cells are driven towards a latent infection in the absence of the ND10 complex.

To determine if the ND10 complex contributes to the silencing of the HHV-6A genome, we used 293T cells that allow quiescent infection and integration with limited lytic replication. Depletion of the three major ND10 components resulted in an increased expression of GFP encoded in the HHV-6A genome. In addition, the major capsid protein U57 was also less efficiently silenced in the absence of the ND10 complex. Even though there was higher expression of the late major capsid protein, we did not observe productive virus replication in these cells. Taken together, our data provides the first insight into the role of ND10 during HHV-6A infection. We could demonstrate that the ND10 complex limits HHV-6A replication and facilitates more efficient silencing of the virus. This could in part explain why HHV-6A is more prone to establishing a quiescent infection than other herpesviruses that dissociate the ND10 complex. The ND10 complex has previously been shown to induce epigenetic modifications on incoming viral genomes such as HSV-1 or Kaposis sarcoma associated herpesvirus [44,45,46]. The epigenetic modification on the HHV-6 genome and the role of the ND10 complex remains elusive and is therefore an exciting avenue for further investigations. Recent studies on HSV-1 by Merkl et al. also suggested involvement of the cellular interferon-inducible protein 16 (IFI16) alone, or in combination with the of ND10 complex, thereby restricting virus replication [47]. In the future, we will set to address the role of cellular factors including IFI16 as well as viral factors in these epigenetic modifications during the establishment of HHV-6 latency.

## Figures and Tables

**Table 1:** List of oligonucleotide primers and probes used for qPCR, mutagenesis, and sequencing.

Name	Sequence (5′→3′)
B2M forward	CCAGCAGAGAATGGAAAGTCAA
B2M reverse	TCTCCATTCTTCAGTAAGTCAACTTCA
B2M probe	ATGTGTCTGGGTTTCATCCATCCGACA
HHV-6 U86 forward	TGTACATGGGCTGTAGGAGTTGA
HHV-6 U86 reverse	ACATCCTCTGCTTCCAATCTACAATC
HHV-6 U86 probe	TTCCGAAGCAAAGCGCACCTGG
HHV-6 U57-P2a-GFP forward	GTTTGTGATCGAAAGTGCAGTAGACGGTTTCCATTTTACTTGTACAGCTCGTCCATGCCG
HHV-6 U57-P2a-GFP reverse	GAGAAACCATACCTTTCCAACTCATTATCGAATCATCCATAGGATCTGGAGCGACCAATT
HHV-6 U57 sequencing forward	CTTTGTTGGAGGAGACGATGG
HHV-6 U57 sequencing reverse	GCCTCTTCACTGTTCATCCAA

## Supplementary Materials

The following are available online at http://www.mdpi.com/1999-4915/10/8/401/s1. Figure S1: Effect of HHV-6 infection on the ND10 complex. Figure S2: Schematic image describes the construction of HHV-6A-U57-p2A-GFP BAC upon en *passant* mutagenesis.

## References

[1] Levy J.A., Ferro F., Greenspan D., Lennette E.T. (1990). Frequent isolation of HHV-6 from saliva and high seroprevalence of the virus in the population. Lancet.

[2] Okuno T., Takahashi K., Balachandra K., Shiraki K., Yamanishi K., Takahashi M., Baba K. (1989). Seroepidemiology of human herpesvirus 6 infection in normal children and adults. J. Clin. Microbiol..

[3] Zerr D.M., Meier A.S., Selke S.S., Frenkel L.M., Huang M.-L., Wald A., Rhoads M.P., Nguy L., Bornemann R., Morrow R.A. (2005). A Population-Based Study of Primary Human Herpesvirus 6 Infection. N. Engl. J. Med..

[4] Yamanishi K., Shiraki K., Kondo T., Okuno T., Takahashi M., Asano Y., Kurata T. (1988). Identification of human herpesvirus-6 as a causal agent for exanthem subitum. Lancet.

[5] Hall C.B., Long C.E., Schnabel K.C., Caserta M.T., McIntyre K.M., Costanzo M.A., Knott A., Dewhurst S., Insel R.A., Epstein L.G. (1994). Human Herpesvirus-6 Infection in Children—A Prospective Study of Complications and Reactivation. N. Engl. J. Med..

[6] McCullers J.A., Lakeman F.D., Whitley R.J. (1995). Human herpesvirus 6 is associated with focal encephalitis. Clin. Infect. Dis..

[7] Luppi M., Marasca R., Barozzi P., Ferrari S., Ceccherini-Nelli L., Batoni G., Merelli E., Torelli G. (1993). Three cases of human herpesvirus-6 latent infection: Integration of viral genome in peripheral blood mononuclear cell DNA. J. Med. Virol..

[8] Arbuckle J.H., Medveczky M.M., Luka J., Hadley S.H., Luegmayr A., Ablashi D., Lund T.C., Tolar J., De Meirleir K., Montoya J.G. (2010). The latent human herpesvirus-6A genome specifically integrates in telomeres of human chromosomes in vivo and in vitro. Proc. Natl. Acad. Sci. USA.

[9] Ward K.N., Leong H.N., Thiruchelvam A.D., Atkinson C.E., Clark D.A. (2007). Human Herpesvirus 6 DNA Levels in Cerebrospinal Fluid Due to Primary Infection Differ from Those Due to Chromosomal Viral Integration and Have Implications for Diagnosis of Encephalitis. J. Clin. Microbiol..

[10] Daibata M., Taguchi T., Nemoto Y., Taguchi H., Miyoshi I. (1999). Inheritance of chromosomally integrated human herpesvirus 6 DNA. Blood.

[11] Osterrieder N., Wallaschek N., Kaufer B.B. (2014). Herpesvirus Genome Integration into Telomeric Repeats of Host Cell Chromosomes. Annu. Rev. Virol..

[12] Pellett P.E., Ablashi D.V., Ambros P.F., Agut H., Caserta M.T., Descamps V., Flamand L., Gautheret-Dejean A., Hall C.B., Kamble R.T. (2012). Chromosomally integrated human herpesvirus 6: Questions and answers. Rev. Med. Virol..

[13] Caselli E., Di Luca D. (2007). Molecular biology and clinical associations of Roseoloviruses human herpesvirus 6 and human herpesvirus 7. New Microbiol..

[14] De Bolle L., Naesens L., De Clercq E. (2005). Update on human herpesvirus 6 biology, clinical features, and therapy. Clin. Microbiol. Rev..

[15] Tavalai N., Stamminger T. (2009). Interplay between Herpesvirus Infection and Host Defense by PML Nuclear Bodies. Viruses.

[16] Everett R.D. (2001). DNA viruses and viral proteins that interact with PML nuclear bodies. Oncogene.

[17] Maul G.G., Guldner H.H., Spivack J.G. (1993). Modification of discrete nuclear domains induced by herpes simplex virus type 1 immediate early gene 1 product (ICP0). J. Gen. Virol..

[18] Ishov A.M., Sotnikov A.G., Negorev D., Vladimirova O.V., Neff N., Kamitani T., Yeh E.T., Strauss J.F., Maul G.G. (1999). PML is critical for ND10 formation and recruits the PML-interacting protein daxx to this nuclear structure when modified by SUMO-1. J. Cell Biol..

[19] Everett R.D., Chelbi-Alix M.K. (2007). PML and PML nuclear bodies: Implications in antiviral defence. Biochimie.

[20] Tavalai N., Stamminger T. (2008). New insights into the role of the subnuclear structure ND10 for viral infection. Biochim. Biophys. Acta (BBA) Mol. Cell Res..

[21] Everett R.D., Murray J. (2005). ND10 Components Relocate to Sites Associated with Herpes Simplex Virus Type 1 Nucleoprotein Complexes during Virus Infection. J. Virol..

[22] Müller S., Dejean A. (1999). Viral Immediate-Early Proteins Abrogate the Modification by SUMO-1 of PML and Sp100 Proteins, Correlating with Nuclear Body Disruption. J. Virol..

[23] Bernardi R., Pandolfi P.P. (2007). Structure, dynamics and functions of promyelocytic leukaemia nuclear bodies. Nat. Rev. Mol. Cell Biol..

[24] Zhong S., Salomoni P., Pandolfi P.P. (2000). The transcriptional role of PML and the nuclear body. Nat. Cell Biol..

[25] Lee H.-R., Kim D.-J., Lee J.-M., Choi C.Y., Ahn B.-Y., Hayward G.S., Ahn J.-H. (2004). Ability of the Human Cytomegalovirus IE1 Protein To Modulate Sumoylation of PML Correlates with Its Functional Activities in Transcriptional Regulation and Infectivity in Cultured Fibroblast Cells. J. Virol..

[26] Tavalai N., Adler M., Scherer M., Riedl Y., Stamminger T. (2011). Evidence for a Dual Antiviral Role of the Major Nuclear Domain 10 Component Sp100 during the Immediate-Early and Late Phases of the Human Cytomegalovirus Replication Cycle. J. Virol..

[27] Korioth F., Maul G.G., Plachter B., Stamminger T., Frey J. (1996). The nuclear domain 10 (ND10) is disrupted by the human cytomegalovirus gene product IE1. Exp. Cell Res..

[28] Saffert R.T., Kalejta R.F. (2007). Human Cytomegalovirus Gene Expression Is Silenced by Daxx-Mediated Intrinsic Immune Defense in Model Latent Infections Established In Vitro. J. Virol..

[29] Cantrell S.R., Bresnahan W.A. (2006). Human Cytomegalovirus (HCMV) UL82 Gene Product (pp71) Relieves hDaxx-Mediated Repression of HCMV Replication. J. Virol..

[30] Preston C.M., Nicholl M.J. (2006). Role of the cellular protein hDaxx in human cytomegalovirus immediate-early gene expression. J. Gen. Virol..

[31] Wallaschek N., Sanyal A., Pirzer F., Gravel A., Mori Y., Flamand L., Kaufer B.B. (2016). The Telomeric Repeats of Human Herpesvirus 6A (HHV-6A) Are Required for Efficient Virus Integration. PLoS Pathog..

[32] Tischer B.K., von Einem J., Kaufer B., Osterrieder N. (2006). Two-step red-mediated recombination for versatile high-efficiency markerless DNA manipulation in *Escherichia coli*. Biotechniques.

[33] Glass M., Everett R.D. (2013). Components of promyelocytic leukemia nuclear bodies (ND10) act cooperatively to repress herpesvirus infection. J. Virol..

[34] Schippers T., Jarosinski K., Osterrieder N. (2015). The ORF012 Gene of Marek’s Disease Virus Type 1 Produces a Spliced Transcript and Encodes a Novel Nuclear Phosphoprotein Essential for Virus Growth. J. Virol..

[35] Kaufer B.B. (2013). Detection of integrated herpesvirus genomes by fluorescence in situ hybridization (FISH). Methods Mol. Biol..

[36] Kaufer B.B., Jarosinski K.W., Osterrieder N. (2011). Herpesvirus telomeric repeats facilitate genomic integration into host telomeres and mobilization of viral DNA during reactivation. J. Exp. Med..

[37] Rens W., Fu B., O’Brien P.C., Ferguson-Smith M. (2006). Cross-species chromosome painting. Nat. Protoc..

[38] Gravel A., Gosselin J., Flamand L. (2002). Human Herpesvirus 6 immediate-early 1 protein is a sumoylated nuclear phosphoprotein colocalizing with promyelocytic leukemia protein-associated nuclear bodies. J. Biol. Chem..

[39] Seeler J.-S., Marchio A., Sitterlin D., Transy C., Dejean A. (1998). Interaction of SP100 with HP1 proteins: A link between the promyelocytic leukemia-associated nuclear bodies and the chromatin compartment. Proc. Natl. Acad. Sci. USA.

[40] Maul G.G., Negorev D., Bell P., Ishov A.M. (2000). Review: Properties and Assembly Mechanisms of ND10, PML Bodies, or PODs. J. Struct. Biol..

[41] Wagenknecht N., Reuter N., Scherer M., Reichel A., Müller R., Stamminger T. (2015). Contribution of the Major ND10 Proteins PML, hDaxx and Sp100 to the Regulation of Human Cytomegalovirus Latency and Lytic Replication in the Monocytic Cell Line THP-1. Viruses.

[42] Isaac A., Wilcox K.W., Taylor J.L. (2006). SP100B, a repressor of gene expression preferentially binds to DNA with unmethylated CpGs. J. Cell. Biochem..

[43] Wilcox K.W., Sheriff S., Isaac A., Taylor J.L. (2005). SP100B is a repressor of gene expression. J. Cell. Biochem..

[44] Lieberman P.M. (2016). Epigenetics and Genetics of Viral Latency. Cell Host Microbe.

[45] Günther T., Schreiner S., Dobner T., Tessmer U., Grundhoff A. (2014). Influence of ND10 Components on Epigenetic Determinants of Early KSHV Latency Establishment. PLoS Pathog..

[46] Knipe D.M. (2015). Nuclear Sensing of Viral DNA, Epigenetic Regulation of Herpes Simplex Virus Infection, and Innate Immunity. Virology.

[47] Merkl P.E., Orzalli M.H., Knipe D.M. (2018). Mechanisms of Host IFI16, PML, and Daxx Protein Restriction of Herpes Simplex Virus 1 Replication. J. Virol..

