# Supplementary material for: The ND10 Complex Represses Lytic Human Herpesvirus 6A Replication and Promotes Silencing of the Viral Genome
Source: Viruses. 2018 Jul 29;10(8):401. doi: 10.3390/v10080401 (PMC6115956; doi:10.3390/v10080401)
Supplement: Supplementary file 1 [file viruses-10-00401-s001.zip › viruses-320414-SI.pdf]

Supplementary Material.

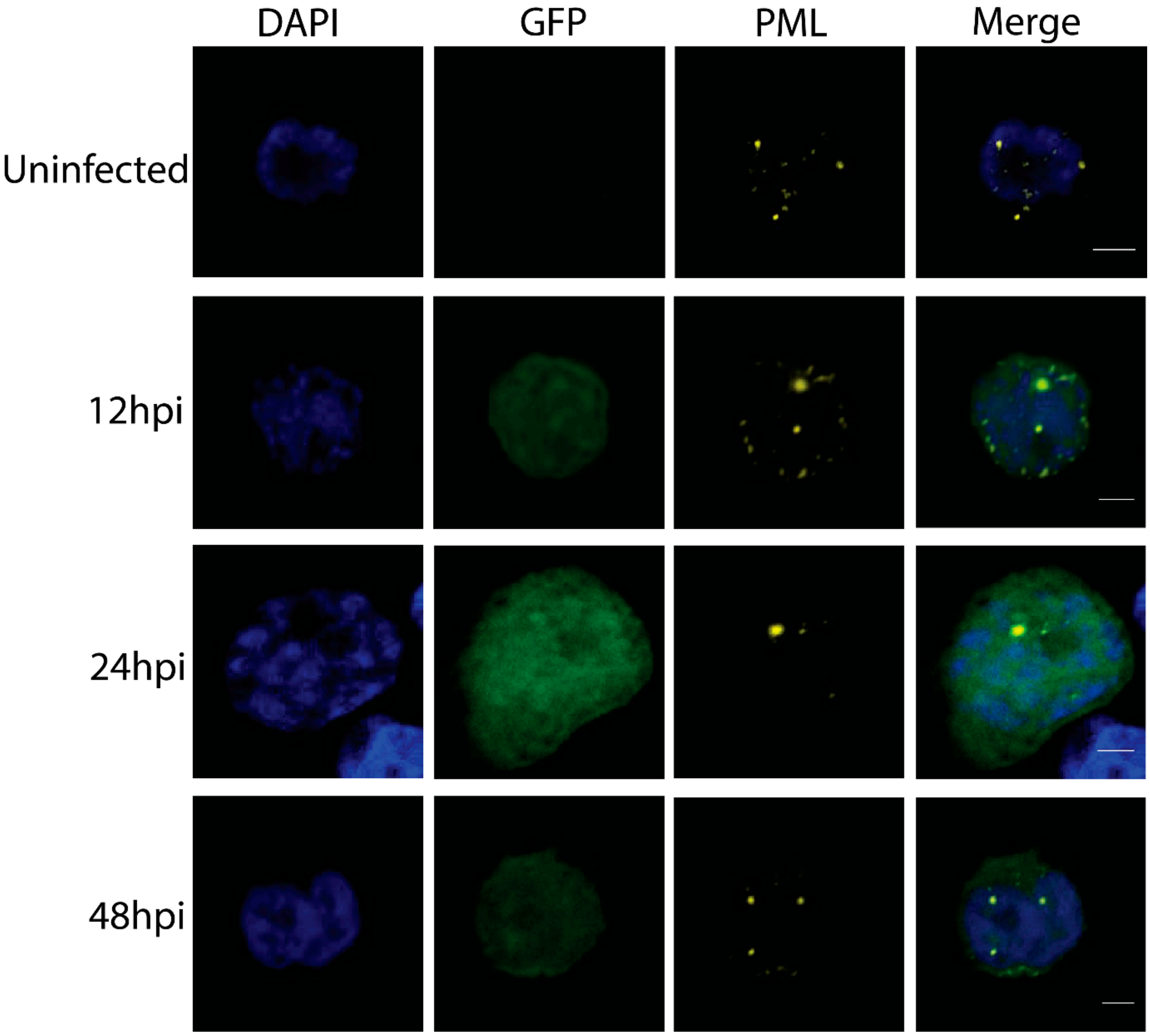

Figure S1. Effect of HHV-6 infection on the ND10 complex. (A) HHV-6A-GFP and mock infected JJHan cells were immunostained for PML (yellow) and analyzed by confocal microscopy. Virus infected cells express GPF (green) and nuclei were stained with DAPI (blue). Representative images are shown for mock and HHV-6A-GFP infected cells at 12, 24 and 48 hours post infection. The scale bars correspond to 3μm.

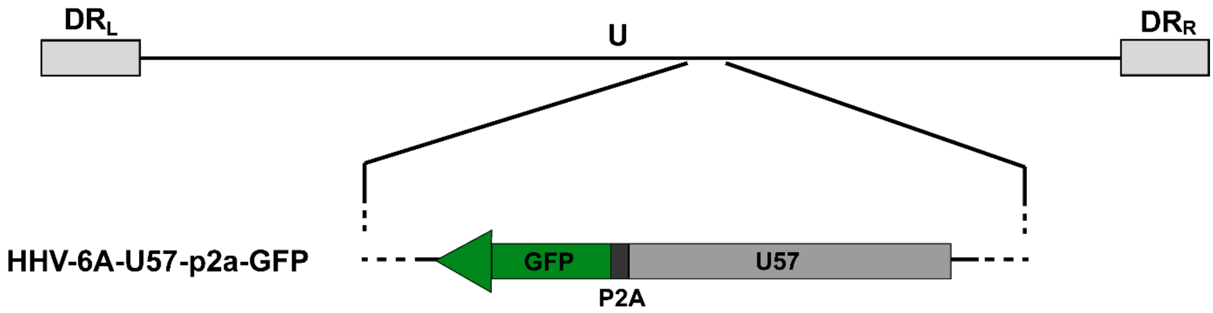

Figure S2. Schematic image describes the construction of HHV-6A-U57-p2A-GFP BAC upon en *passant* mutagenesis.
